# The use of artificial intelligence for automatic analysis and reporting of software defects

**DOI:** 10.3389/frai.2024.1443956

**Published:** 2024-12-11

**Authors:** Mark Esposito, Saman Sarbazvatan, Terence Tse, Gabriel Silva-Atencio

**Affiliations:** ^1^Hult International Business School, Cambridge, MA, United States; ^2^Berkman Klein Center for Internet and Society at Harvard University, Cambridge, MA, United States; ^3^École des ponts ParisTech (ENPC), Marne-la-Vallée, France; ^4^Universidad Latinoamérica de Ciencia y Tecnología (ULACIT), San José, Costa Rica

**Keywords:** artificial intelligence, software development, automation testing, software defect, software failure

## Abstract

The COVID-19 pandemic marked a before and after in the business world, causing a growing demand for applications that streamline operations, reduce delivery times and costs, and improve the quality of products. In this context, artificial intelligence (AI) has taken a relevant role in improving these processes, since it incorporates mathematical models that allow analyzing the logical structure of the systems to detect and reduce errors or failures in real-time. This study aimed to determine the most relevant aspects to be considered for detecting software defects using AI. The methodology used was qualitative, with an exploratory, descriptive, and non-experimental approach. The technique involved a documentary review of 79 bibliometric references. The most relevant finding was the use of regression testing techniques and automated log files, in machine learning (ML) and robotic process automation (RPA) environments. These techniques help reduce the time required to identify failures, thereby enhancing efficiency and effectiveness in the lifecycle of applications. In conclusion, companies that incorporate AI algorithms will be able to include an agile model in their lifecycle, as they will reduce the rate of failures, errors, and breakdowns allowing cost savings, and ensuring quality.

## 1 Introduction

Artificial Intelligence (AI) is not a technology far from everything that surrounds us in our daily lives: robots, computers that understand our language, and autonomous cars, among others, are part of the future that we saw on television and that we now have in our present (Kacena et al., 2024; Finlay and Dix, 1996).

AI in software quality assurance has brought great benefits to companies, helping in tasks such as predicting and locating defects, sequence learning, code cloning, and many other functions. Correctly using these technologies has resulted in software companies reducing the effort, time, and costs required for defect discovery and resolution (Satapathy et al., 2020).

In recent years, there has been a strong desire by engineers and companies to automate as much as possible and to use the latest trends in technology. However, this focus often overlooks the potential side effects. Despite this, AI is increasingly being applied to autonomously detect and analyze defects. In the early days of software development, people wrote program code and found defects as they used it during development (Acemoglu and Restrepo, 2018; Rodríguez-Pérez et al., 2020). Later, to improve the quality of applications, more thorough testing was incorporated to detect defects as early as possible (Xu et al., 2014). Consequently, as the lines of code increased, the testing and verification processes became longer and more complex, even causing errors or omissions that were performed manually (Nagaria and Hall, 2020).

The processes for analyzing and detecting faults or failures in information applications are highly time-consuming. Kushwaha and Misra (2008) identified that the tests in these technological platforms must be repeated several times, in order for the developers to identify the root cause of the defect, an arduous job that does not guarantee that the failure will be fully corrected. Thus, this study aims to identify the main techniques that can be used to reduce the failure rate for these technological solutions automatically through the use of AI.

If the process of analysis and failure detection could be automated, companies would experience continuous improvement. Automating tasks related to planning, execution, control, and monitoring in application coding would reduce the time and costs, while enhancing product quality, as many of these tasks would be performed in an automated way. Gonçalves et al. (2017) identified that, by freeing collaborators from software quality review tasks, this knowledge, experience, and expertise could be used in new processes and procedures of new applications. However, automation also has its limitations, Skripchuk et al. (2022) identified that if machine learning (ML) algorithms are not trained with sufficient statistical data that represents the universe, undesired results can be obtained, generating failures in the applications that can have an impact in different areas of the organization.

## 2 Literature review

The implementation of AI is based on a measurable characteristic and its impact on business needs. A clear example is the projects that use AI to automate many manual processes, providing a solution to the users in charge of these tasks. However, there are also AI projects that need constant justifications to keep them running when they could be replaced by other projects that perform more functions in a better way (Moore, 2019).

Before continuing, let us define the concept of AI. Suleimenov et al. (2020) cataloged it from the field of computer science as a discipline and a set of cognitive and intellectual capabilities expressed by computer systems or combinations of algorithms whose purpose is the creation of machines that mimic human intelligence to perform tasks, and that can improve as they collect information.

In the field of AI, different methods can be used to efficiently manage data. Robotic process automation (RPA) tries to reduce human intervention mainly by interacting with high-level applications, which are the graphical interface layers (Madakam et al., 2019), i.e., it is an application that emulates the real interaction that a human would have with conventional computer applications. Whereas, ML is an application of AI that allows systems to learn and improve automatically by using data patterns; once these patterns are detected, ML adjusts the program’s actions using algorithms (Baştanlar and Özuysal, 2014; Bi et al., 2019).

To understand how process automation works using AI, one must know the four principles of intelligent automation: Thinking and Learning, Vision, Language, and Execution (Tyagi et al., 2021). The first principle is based on where technologies such as ML, data visualization, and big data analytics are used (Dohn et al., 2022). Second is vision, which allows computers to analyze and process activities such as optical character recognition (OCR), intelligent character recognition (ICR), video and image analysis, and biometric data analysis and processing (Li and Shi, 2018). Language is the third principle that includes technologies such as Chatbots (conversations with intelligent robots) and unstructured information management (UIM), as well as sentiment and speech analysis (Bornet et al., 2021). Finally, the execution is the principle based on low-code (no-code) technologies, allowing individuals without software development knowledge to create applications or automate repetitive processes using RPA (Sahay et al., 2020).

Undoubtedly, the quality of the application development methodology has essential aspects that must be considered throughout its lifecycle, to ensure the requirements and provide value to the company (Saran, 2023). Barstow (1988), as part of his study, indicates that AI brings new functionalities in the software development process as it provides functions that automate the intellectual capacity of the individual, such as automated processes, Chatbots, and autonomous robots.

AI applied to software testing is evolving, altering the testing landscape by taking this process to another level where it will no longer rely so much on human reasoning. It focuses on facilitating the lifecycle by applying logic, problem-solving, and ML to solve tedious tasks related to software testing and its limitations. AI reviews recent code changes, code coverage, and other software testing metrics to decide which tests to run and when to run them, helping developers focus on other, more valuable tasks for the benefit of the company or organization (Dong et al., 2022; Ramalho et al., 2020).

The use of AI in test scenario optimization is projected to grow in every facet of creative technology due to the increasing number of applications we use daily (Jain, 2023). AI applied to software testing aims to help organizational teams develop and test their code efficiently and effectively and create higher-quality software faster (Khaliq et al., 2022). Januszewski et al. (2021) and Lamberton et al. (2017) agree that AI, through applications such as RPA, can provide the ability to automate business processes, through repetitive tasks, along with the ability to integrate with other management information systems and facilitate the recording, organization, review and reporting of processes, activities, and tasks that impact the business.

Seth and Bagalkoti (2023) identified the ability of RPA to automate business, allowing to improve efficiency and effectiveness in the operation, since it facilitated the generation of automatic reports denoting defects and failures in the software, this is because they had a roadmap that outlined the methodology used by developers when implementing a technological solution.

An important aspect to consider when evaluating the ability of automated solutions to identify potential flaws in software development is the consideration of potential cyber vulnerabilities in development environments. Alfadel et al. (2023) found that the use of supervised and unsupervised algorithms allows the identification of programming code information circulating on the dark web. This allows companies to detect these codes and be notified of possible vulnerabilities in their online infrastructure, helping to predict and address these flaws promptly. Horawalavithana et al. (2019) identified that the use of AI also allows for identifying vulnerabilities that are published as code flaws in technological platforms through comments in social networks, which allows for detecting vulnerabilities and exposures promptly.

A possible hypothesis that could arise around the use of AI is whether the technology can identify possible faults or errors in the code through automated tests based on supervised algorithms. Singh et al. (2016) posit that to achieve this goal, algorithms must be trained with valid old records, which allows for establishing the basis of coding and determining the root cause of a defect without supervision. In the year 2023, there were already technological platforms in the industry that provided automated tests for the verification of coding errors in web pages: (1) Selenium, is one of the leading platforms in this area, but it has some limitations such as the lack in handling dynamic web pages, reducing its responsiveness and accuracy in the results (Alsuwailem and Alharbi, 2023), and (2) Cypress simplifies asynchronous testing by executing tests only after everything necessary is loaded in the web application. It employs a test-driven development (TDD) methodology and uses a Page Object Model (POM) framework, which provides high profitability. This approach increases the number of functions covered by the tests, improves execution speed by 21%, and reduces complexity of automation by 31% (Mobaraya and Ali, 2019).

Currently, as different automation environments could be used to generate reports that are useful during the development lifecycle of an application, Yatskiv et al. (2019) and Li et al. (2020) agree that RPA can be a powerful tool for the automation of software testing through a user-friendly graphical interface. This would allow the establishment of standardized processes and procedures, leading the organization toward a methodology for the design, planning, development, and implementation of applications, thus reducing the common problems in coding, but to achieve this goal, it is necessary to create the installed base (viable old codes) that will require a cost–benefit analysis by the company. The software testing stage requires an even more significant amount of time and effort than the software development stage, which is why it is vital for achieving the company’s objectives, as it guarantees the robustness of the software (Kushwaha and Misra, 2008).

Software development involves more than just knowing how to develop an application; it encompasses the entire software development lifecycle. This includes steps such as requirements elicitation, application design, creating the working software, testing, and many more steps. However, automating the analysis of failed tests would allow companies to reduce human intervention by performing these tasks using AI (Mauro, 2023).

AI allows companies to automate repetitive processes, which is very useful when autonomously reporting defects in task management tools such as JIRA. This allows staff to focus on different areas, as AI, together with ML, enhances RPA, speeding up decision-making and reducing the risk of errors (UIPath, 2023).

It is worth noting that there are relevant differences between the technologies because RPA focuses more on “doing.” At the same time, AI and ML are more concerned with “thinking and learning”; another difference is that RPA focuses primarily on processes. In contrast, AI and ML focus on data quality and how these help in good application development. RPA uses structured and logical inputs, while AI uses unstructured inputs and develops logical fields (NICE, 2023).

Use cases can be created through the processes performed manually by users for defect reporting to allow RPA to automate this task. While AI and ML analyze the root cause of defects, RPA can take care of automatic defect reporting by first analyzing whether any similar defects are reported in the task management tool to avoid creating duplicate tickets (Bots, 2023). Because the uses or functions of the software are different within each company, and what is essential to one company is not necessary to another, each organization should automate its processes based on its usage manuals or policies established within the organization (Voss, 1985).

With increased competition among companies, the demand for software developers is constantly growing, resulting in an increase in the number of lines of code that must be analyzed and tested before they are considered optimal for delivery (Baddoo et al., 2006). Lodge (2023) identified that software developers spend 35% of their productive time on software testing.

With the imminent and forced technological update brought by the COVID-19 pandemic, coupled with the lack and difficulty of hiring qualified personnel, companies are increasingly forced to do more with the staff they have. This leads to the subject of this research, looking at how to automate those monotonous tasks. Aspects such as regression testing, log files automated test, and data validation method, through technologies such as AI, thus, manage to free up quality time for jobs that require more human input from the staff.

Although it might seem like common sense, implementing AI in software testing must address a major bottleneck: the need for an oracle. This mechanism defines what is right and what is wrong. AI is rarely used in bug detection due to the problem of automating the prophet; the only exception to this rule is regression testing, in which expected results can be analyzed and determined based on previous versions of the system or software (Lima et al., 2020).

Finally, all this saving of time means more quality time that developers and the rest of the team can allocate to more intellectually challenging work and any other task aimed at meeting the needs of customers or organizations. However, it should be noted that, despite the many benefits that may bring the automation of software testing, if such automation is based on poorly designed processes, this will only potentiate errors and cause chaos and confusion for the team.

Finally, and most importantly, this new wave of thinking and implementation will allow the team to be free from repetitive and tedious tasks. It will give breathing room and creative freedom to other, more innovative ideas to benefit organizations that risk investing in these technologies.

## 3 Methodology

In the context of this study, a methodology that amalgamates a narrative and critical literature review with an exploratory and descriptive qualitative approach was implemented. The objective was to determine the most relevant aspects to be considered within AI that enable automatic analysis and reporting of software defects.

As a starting point, a narrative and critical review of previously existing literature in the field of automatic analysis and reporting of software defects was conducted to develop a solid conceptual framework based on previous research (Creswell et al., 2007). The methodology relied on a deductive line of reasoning to logically and productively structure the proposal of this study.

Consequently, it allowed a deep dive into the automatic analysis and reporting of software defects and the emerging trends in this field, focusing on the coexistence and evolution of AI automation testing to detect software defects, based on the approaches cited (Mariani et al., 2023).

To this end, a bibliometric review was carried out that included the review of scientific articles from 2013 to 2024, from here 79 bibliographic references highlighted to the topic of the study were selected.

Subsequently, the documentary review was conducted through searches in electronic databases, such as Google Scholar, Web of Science, Emerald, Scopus, Science Direct, and EBSCO host, and consultation of websites of recognized authors in the field. These were carried out in Spanish and English, using specific search criteria, incorporating keywords such as “artificial intelligence,” “automation testing,” “software defects,” “software development,” “software failures,” “quality assurance,” and “quality control.”

Once the sources were compiled, the search for scientific articles was limited to finding 79 articles among the scientific databases consulted for the established time interval.

Subsequently, the 79 summaries of each research were read, which allowed the classification of the research into the categories of literature review and use cases. Then, by counting words and phrases, we were able to identify the aspects with the greatest repetition in the studies that stood out as relevant to the topic addressed in this research.

Finally, with all the information gathered, we proceeded to identify and infer the characteristics, highlighting the controversies, main conversations, and threats associated with using AI for automatic analysis and reporting of software defects. This analysis is supported by notable researchers such as Khaliq et al. (2022), Ricca et al. (2021), and Job (2021), who highlight the growth and diversity of approaches in the use of AI for automation testing software.

## 4 Results

The phrase “artificial intelligence” appeared in 95% of the searches performed, which when combined with the word “automation testing” was reduced to 75% of the results obtained. In contrast, when combinations of “artificial intelligence” + “automation testing” + “software development” with “software defects” were performed, the results were reduced to 0.0014%, suggesting an opportunity for studies related to the use of artificial intelligence for automated software testing for defect detection. Other relevant descriptors were also identified, such as “regression testing,” “automated test,” “data validation,” “binary classification,” “machine learning algorithms,” “RPA,” and “API,” which allowed inferring the existence of a state of the art on the use of artificial intelligence for automatic analysis and reporting of software defects in a comprehensive manner and from a multidisciplinary perspective. Table 1 presents the results by year of the articles related to the subject of the study.

**Table 1:** Results of the search for the object of study.

Year	Items	Percentage
2013	1	1.27%
2014	0	0.00%
2015	0	0.00%
2016	1	1.27%
2017	4	5.06%
2018	6	7.59%
2019	8	10.13%
2020	12	15.19%
2021	15	18.99%
2022	20	25.32%
2023	12	15.19%
2024	0	0.00%

As shown in Table 2, the object of study acquired great relevance as of 2019, mainly driven by the commercial boom of artificial intelligence. Khakurel et al. (2018) and Krittanawong (2018) agree that AI has taken a preponderant role in the digital era since it has allowed the acceleration of the digital transformation processes in organizations facing an uncertain future, while Vaishya et al. (2020) state that the use of AI is a fundamental element for the survival of organizations after the impacts caused by the COVID-19 pandemic.

**Table 2:** Scientific studies identified.

Type of study	Items	Percentage
Literature review (Empirical)	37	46.84%
Case studies	42	53.16%

Table 2 summarizes the selected studies and the standard for identifying patterns and emerging trends when using AI for automatic analysis and reporting of software defects.

Table 2 shows two significant categories associated with the theme, the details of which are set out below:

The literature review, which includes empirical studies, has a 46.84% representation in the results obtained. However, when reviewing the content of the studies in detail, by counting words and phrases, the following results were identified and obtained (see Table 3).

**Table 3:** Relevant findings in the literature review.

Topic	Number of matches	Percentage
Regression testing	37	36.27%
Log files automated test	30	29.41%
Data validation method and model	20	19.61%
Binary classification	15	14.71%

The results highlight the studies that take as their main interest the use of regression algorithms for automatic analysis and notification of software defects with 36.27% importance. In second position, with 29.41% is the use of automated testing through RPA to review the logs of the log files generated by the applications. Then, in third position (19.61%), is the use of methods and models for data validation with the help of AI. Finally, 14.71% is the use of binary data classification algorithms. An important aspect to highlight is that the studies agree on the use of algorithms as a tool for predicting future patterns, but with the caveat that to obtain this result, it is necessary to have a statistically significant data sample, which allows training the models and avoiding an unexpected result.

Case studies (42 studies, 53.16%): This category is highlighted within the findings as developers are on a quest for detailed knowledge of uses, applications, experiences, and lessons learned in the field surrounding the use of AI for automatic analysis and software defect notification. The following have been documented for academic and scientific purposes. These have been documented for academic and scientific purposes (see Table 4).

**Table 4:** Relevant findings in the case studies.

Topic	Number of matches	Percentage
Regression testing	69	31,5%
Log files automated test	53	24.5%
Data validation method and model	38	17.5%
Machine learning algorithms	24	11%
RPA	20	9%
API	14	6.5%

The results are grouped into six aspects: (1) regression testing (31.5%), (2) log files automated test (24.5%), (3) data validation method and model (17.5%), indicating the AI applications that are being documented in science and that allow us to see the trend that this technology is taking for the automatic analysis of software defects as main aspects to be considered, (4) machine learning algorithms (11%), (5) RPA (9%), and (6) API (6.5%), which provides us with evidence of the most common aspects as models and processes in AI that allow us to detect and report a software defect. All categories document the impact of the use of mathematical models to predict data patterns that can be efficiently and effectively identified by AI, identifying lessons learned that seek to document these aspects as implications in a timely manner.

In summary, the use of AI for automated software testing for defect detection has advanced in the field of software engineering. In recent years, there have been emerging studies that seek evidence in the field of how AI technology can contribute to the efficiency and effectiveness of processes. However, from the results obtained in the bibliometric review conducted on the subject, together with recognized authors and the expert judgment of researchers, it has been identified that it is still an area with great opportunities for exploration since the studies are only focused on applications such as ML and RPA, through prediction models such as regression testing and log files automated test, which generates a huge potential for transformation for the incorporation of AI in the lifecycle of applications. These findings highlight an opportunity for scientific studies to address these issues as part of the digital acceleration processes.

## 5 Discussion

The results obtained highlight the main trends in the use of AI for software defect detection, the relevant categories being the literature review and case studies, where regression testing, the log files automated test model, and the data validation method and model stand out and coincide in both categories. Therefore, the importance and relevance of these findings are expanded in this section with the following articles.

Jyolsna and Anuar (2022) in their study used the automatic testing functionality of the Cypress Test Runner in a web environment; obtaining as results the execution of software tests and generation of a report of the positive and negative results of the tests in real-time. However, the study highlights the complexity of the automatic testing process. Without a data validation method and model in the development environment, it becomes impossible to establish a baseline to guide the testing process. This can lead to multiple validation paths, potentially containing untrained data, and result in different versions of tests and reports due to the lack of a proper methodology (regression testing and log files automated test).

Pelivani et al. (2022) emphasize the importance of having aspects such as regression testing, log files automated test, and data validation method, so that the Cypress AI algorithms can function correctly in the generation of information and report generation. The lack of this pattern will generate an error log in the automated tests, due to the lack of a standard. Satapathy et al. (2020) identified that when an error occurs in the execution of the automated test, the Cypress-Failed-Log application generates a log in JavaScript Object Notation (JSON) format with the data associated with the error identified in the test, being a functionality that adds value to the software testing process.

de Silva et al. (2023) identified in their field study on the Cypress application, that the application at the moment of having the normalized data, uses algorithms based on natural language processing (NLP), normalizing the content being created within the JSON file, through the sintering of the data, using the words and phrases that are not necessary.

Ahmadi (2023) emphasizes that any automatic software testing process must start with the training process of the data validation method and model; in the case of Cypress-Failed-Log, training is performed from the log files (JSON) generated, which is then converted into information that is parameterized through a token.

After the creation of the token, the entities to be used are created, which contain the information and categories of error classification; which are entered as part of the process for error analysis (Trad, 2023), being available during the analysis process, evaluation, and reporting of software defects at the time of the study, being available during the analysis process an opportunity for the development of future study in the field of self-analysis of software defects.

An alternative solution to the lack of automated platforms for the analysis, evaluation, and reporting of software defects, would be the delivery of the software coding done by the developers as a source (baseline) for the establishment of a roadmap that would allow the creation of a version comparison in the solution to be designed. Bui et al. (2020) mention that AI has the capability and potential to search within a repository all the changes in the coding of the software programming performed; and from a baseline of the coding provided by the company, identify the variations in real-time using an automatic analysis and testing process.

Seth and Bagalkoti (2023) argue that an RPA platform could access a repository with the details of the reported bugs (data), and from here it would run the comparison process to identify the defect in the software and generate the report with the identified finding. However, this functionality is not yet available for software engineering organizations, remaining only as an assumption or proposal to be evaluated by future researchers.

## 6 Conclusion

### 6.1 Context

Currently, the business sector is under strong pressure to be increasingly competitive in the digital landscape. The use of AI will help create more agile organizations by reducing the mechanical activities associated with quality processes for products, goods, and services.

### 6.2 Findings

However, the limitation to incorporating an agile mindset in software defects will depend on the data management models inside the culture. Examples of these aspects are the use of regression tests and automated log files, which can contribute to the reduction of errors. Unfortunately, the lack of methodologies to implement a software defects protocol is currently a challenge to several companies, since there is no standard roadmap for reporting software bugs and defects that can serve as a collaborative knowledge to the industry.

The lack of this methodological standard has led to the generation of research studies that seek to identify best practices to be applied to the business sector. However, in academia, common thoughts are emerging that promote the use of mathematical models based on AI, such as regression tests or mathematical procedures for automated processes.

### 6.3 Recommendations

The following recommendations are proposed based on the results obtained:

1. Obtaining, validating, and arranging the data as a representative sample of the functionality to be evaluated.
2. Verify and validate the training model (supervised or unsupervised) from the data model, to obtain the most accurate results possible.
3. Establish a methodology for documenting the results of the software tests, to document failures, errors, and faults.
4. Establish within the methodology a roadmap to identify the root cause of a possible failure or malfunction.

### 6.4 Future research

Finally, from the findings identified in this study related to the use of AI for software defects, it was possible to identify the use and proliferation of this science in the last 5 years, through practical applications in the fields of ML and RPA, which allow the identification, analysis, testing, and reporting of errors and faults that have been identified in previous scenarios, improving costs, and ensuring quality.

In the area of future research lines, the use of AI for software defect notification will be explored in greater detail, through field tests that allow for generating a greater number and variety of case studies.

## Data Availability

The original contributions presented in the study are included in the article/supplementary material, further inquiries can be directed to the corresponding author.

## References

[ref1] Acemoglu D. Restrepo P. (2018). Artificial intelligence, automation, and work (the economics of artificial intelligence: An agenda). Chicago, IL: University of Chicago Press, 197–236.

[ref2] Ahmadi S. (2023). A tokenization system for the Kurdish language. ACL Anthology. Available online at: https://aclanthology.org/2020.vardial-1.11

[ref3] Alfadel M. Costa D. E. Shihab E. (2023). Empirical analysis of security vulnerabilities in python packages. Empir. Softw. Eng. 28:59. doi: 10.1007/s10664-022-10278-4

[ref4] Alsuwailem G. Alharbi O. (2023). "utilizing machine learning for predicting software faults through selenium testing tool," international journal of computations. Informat. Manufact. 3, 13–27.

[ref5] Baddoo N. Hall T. Jagielska D. (2006). Software developer motivation in a high maturity company: a case study. Software Process 11, 219–228. doi: 10.1002/spip.265

[ref6] Barstow D. (1988). Artificial intelligence and software engineering. Exploring Artif. Intel., 641–670. doi: 10.1016/B978-0-934613-67-5.50020-4

[ref7] Baştanlar Y. Özuysal M. (2014). “Introduction to machine learning” in miRNomics: MicroRNA biology and computational analysis, Humana Press. 105–128. 10.1007/978-1-62703-748-8_7 24272434

[ref8] Bi Q. Goodman K. E. Kaminsky J. Lessler J. (2019). What is machine learning? A primer for the epidemiologist. Am. J. Epidemiol. 188, 2222–2239. doi: 10.1093/aje/kwz189, PMID: 31509183

[ref9] Bornet P. Barkin I. Wirtz J. (2021). Intelligent automation: Welcome to the world of hyperautomation: Learn how to harness artificial intelligence to boost business & make our world more human. World of Hyperautomation.

[ref10] Bots C. (2023). The difference between robotic process automation and artificial intelligence. CFB Bots. Available online at: https://cfb-bots.medium.com/the-difference-between-robotic-process-automation-and-artificial-intelligence-4a71b4834788

[ref11] Bui X. Nguyen H. Choi Y. Nguyen-Thoi T. Zhou J. Dou J. (2020). Prediction of slope failure in open-pit mines using a novel hybrid artificial intelligence model based on decision tree and evolution algorithm. Sci. Rep. 10:9939. doi: 10.1038/s41598-020-66904-y, PMID: 32555284 PMC7303121

[ref12] Creswell J. W. Hanson W. E. Clark Plano V. L. Morales A. (2007). Qualitative research designs: selection and implementation. Couns. Psychol. 35, 236–264. doi: 10.1177/0011000006287390

[ref13] de Silva D. Samarasekara H. M. P. P. K. H. de Silva L. S. M. de Silva W. L. P. Methmini P. K. H. Wijewickrama G. R. P. S.. (2023). Evaluating the effectiveness of different software testing frameworks on software quality. Research Square. doi: 10.21203/rs.3.rs-2928368/v1

[ref14] Dohn N. B. Kafai Y. Mørch A. Ragni M. (2022). Survey: artificial intelligence, computational thinking and learning. KI-Künstliche Intelligenz 36, 5–16. doi: 10.1007/s13218-021-00751-5

[ref15] Dong H. Falis M. Whiteley W. Alex B. Matterson J. Ji S.. (2022). Automated clinical coding: what, why, and where we are? NPJ Dig. Med. 5:159. doi: 10.1038/s41746-022-00705-7, PMID: 36273236 PMC9588058

[ref16] Finlay J. Dix A. (1996). An introduction to artificial intelligence. Boca Raton, FL: CRC Press.

[ref17] Gonçalves W. F. de Almeida C. B. de Araújo L. L. Ferraz M. S. Xandú R. B. de Farias I. (2017). "The influence of human factors on the software testing process: the impact of these factors on the software testing process," in 2017 12th Iberian conference on information systems and technologies (CISTI): IEEE, pp. 1–6.

[ref18] Horawalavithana S. Bhattacharjee A. Liu R. Choudhury N. Hall L. O. Iamnitchi A., (2019). "Mentions of security vulnerabilities on Reddit, twitter and Github," in IEEE/WIC/ACM international conference on web intelligence, pp. 200–207.

[ref19] Jain R. (2023). 23 Software Testing Trends To Look Out For In 2023. Lambdatest. Available online at: https://www.lambdatest.com/blog/software-testing-trends/

[ref20] Januszewski A. Kujawski J. Buchalska-Sugajska N. (2021). Benefits of and obstacles to RPA implementation in accounting firms. Procedia Comput. Sci. 192, 4672–4680. doi: 10.1016/j.procs.2021.09.245

[ref21] Job M. A. (2021). Automating and optimizing software testing using artificial intelligence techniques. Int. J. Adv. Comput. Sci. Appl. 12:120571. doi: 10.14569/IJACSA.2021.0120571

[ref22] Jyolsna J. Anuar S. (2022). Modern web automation with cypress. Io. Open Int. J. Informatics 10, 182–196. doi: 10.11113/oiji2022.10n2.229

[ref23] Kacena M. A. Plotkin L. I. Fehrenbacher J. C. (2024). The use of artificial intelligence in writing scientific review articles. Curr. Osteoporos. Rep. 22, 115–121. doi: 10.1007/s11914-023-00852-0, PMID: 38227177 PMC10912250

[ref24] Khakurel J. Penzenstadler B. Porras J. Knutas A. Zhang W. (2018). The rise of artificial intelligence under the lens of sustainability. Technologies 6:100. doi: 10.3390/technologies6040100

[ref25] Khaliq Z. Farooq S. U. Khan D. A. (2022). Artificial intelligence in software testing: Impact, problems, challenges and prospect. arXiv. doi: 10.48550/arXiv.2201.05371

[ref26] Krittanawong C. (2018). The rise of artificial intelligence and the uncertain future for physicians. Eur. J. Intern. Med. 48, e13–e14. doi: 10.1016/j.ejim.2017.06.017 28651747

[ref27] Kushwaha D. S. Misra A. K. (2008). Software test effort estimation. ACM Sigsoft Software Eng. Notes 33, 1–5. doi: 10.1145/1360602.1361211

[ref28] Lamberton C. Brigo D. Hoy D. (2017). Impact of robotics, RPA and AI on the insurance industry: challenges and opportunities. J. Finan. Perspect. 4. Available at: https://ssrn.com/abstract=3079495

[ref29] Li X. Shi Y. (2018). "Computer vision imaging based on artificial intelligence," International conference on virtual reality and intelligent systems (ICVRIS), pp. 22–25.

[ref30] Li J. Ulrich A. Bai X. Bertolino A. (2020). Advances in test automation for software with special focus on artificial intelligence and machine learning. Softw. Qual. J. 28, 245–248. doi: 10.1007/s11219-019-09472-3

[ref31] Lima R. da Cruz A. Ribeiro J. (2020). "Artificial intelligence applied to software testing: A literature review," in 2020 15th Iberian Conference on Information Systems and Technologies (CISTI). pp. 1–6.

[ref32] Lodge M. (2023). Software testing is tedious. AI can help. Havard Business Review. Available online at: https://hbr.org/2021/02/software-testing-is-tedious-ai-can-help.

[ref33] Madakam S. Holmukhe R. M. Jaiswal D. K. (2019). The future digital work force: robotic process automation (RPA). JISTEM 16:6001. doi: 10.4301/S1807-1775201916001erratum

[ref34] Mariani M. Machado I. Nambisan S. (2023). Types of innovation and artificial intelligence: a systematic quantitative literature review and research agenda. J. Bus. Res. 155:113364. doi: 10.1016/j.jbusres.2022.113364

[ref35] Mauro J. (2023). Intelligent Automation. AuraQuantic. Available online at: https://www.auraquantic.com/what-is-intelligent-automation/

[ref36] Mobaraya F. Ali S. (2019). Technical analysis of selenium and cypress as functional automation framework for modern web application testing," in 9th international conference on computer science.

[ref37] Moore A. (2019). When AI becomes an everyday technology. Harv. Bus. Rev. 7. Available at: https://hbr.org/2019/06/when-ai-becomes-an-everyday-technology

[ref38] Nagaria B. Hall T. (2020). How software developers mitigate their errors when developing code. IEEE Trans. Softw. Eng. 48, 1853–1867. doi: 10.1109/TSE.2020.3040554

[ref39] NICE. (2023). AI and RPA: What is the Difference and Which is Best for Your Organization? NICE Systems. Available online at: https://www.nice.com/rpa/rpa-guide/rpa-ai-and-rpa-whats-the-difference-and-which-is-best-for-your-organization/

[ref40] Pelivani E. Besimi A. Cico B. (2022). "A comparative study of UI testing framework," 11th Mediterranean Conference on Embedded Computing (MECO), pp. 1–5.

[ref41] Ramalho A. Souza J. Freitas A. (2020). The use of artificial intelligence for clinical coding automation: a bibliometric analysis. Int. Symp. Distributed Comput. Artif. Intel. 1237, 274–283. doi: 10.1007/978-3-030-53036-5_30

[ref42] Ricca F. Marchetto A. Stocco A. (2021). "Ai-based test automation: A grey literature analysis," in 2021 IEEE International Conference on Software Testing, Verification and Validation Workshops (ICSTW), pp. 263–270.

[ref43] Rodríguez-Pérez G. Robles G. Serebrenik A. Zaidman A. Germán D. M. Gonzalez-Barahona J. M. (2020). How bugs are born: a model to identify how bugs are introduced in software components. Empir. Softw. Eng. 25, 1294–1340. doi: 10.1007/s10664-019-09781-y

[ref44] Sahay A. Indamutsa A. Di Ruscio D. Pierantonio A. (2020). "Supporting the understanding and comparison of low-code development platforms," in 2020 46th Euromicro Conference on Software Engineering and Advanced Applications (SEAA), pp. 171–178.

[ref45] Saran C. (2023). The Art of developing happy customers: Artificial Intelligence is playing a growing role in modern software development. ComputerWeekly. Available online at: https://www.computerweekly.com/feature/The-art-of-developing-happy-customers.

[ref46] Satapathy S. Jena A. Singh J. Bilgaiyan S. Mahapatra S. Mishra S. (2020). Usage of machine learning in software testing (automated software engineering: a deep learning-based approach), Springer. 39–54.

[ref47] Seth S. Bagalkoti V. (2023). JIRA Report Extraction. Jaypee University of Information Technology, Solan, H.P. Available online at: http://ir.juit.ac.in:8080/jspui/jspui/handle/123456789/7226

[ref48] Singh A. Thakur N. Sharma A. (2016). "A review of supervised machine learning algorithms," in 2016 3rd International Conference on Computing for Sustainable Global Development (INDIACom). pp. 1310–1315.

[ref49] Skripchuk J. Shi Y. Price T. (2022). "Identifying common errors in open-ended machine learning projects," in Proceedings of the 53rd ACM Technical Symposium on Computer Science Education, pp. 216–222.

[ref50] Suleimenov I. E. Vitulyova Y. S. Bakirov A. S. Gabrielyan O. A. (2020). "Artificial intelligence: what is it?," Proceedings of the 2020 6th International Conference on Computer and Technology Applications, pp. 22–25.

[ref51] Trad T. (2023). Integration testing for enterprise web applications. Politecnico di Torino. Available online at: http://webthesis.biblio.polito.it/id/eprint/28677

[ref52] Tyagi A. K. Fernandez T. F. Mishra S. Kumari S. (2021). Intelligent automation Systems at the Core of industry 4.0. Adv. Intel. Syst. Comput., 1–18. doi: 10.1007/978-3-030-71187-0_1

[ref53] UIPath. (2023). AI and RPA Whitepaper – AI Center. UIPath. Available online at: https://www.uipath.com/resources/automation-whitepapers/bringing-power-ai-rpa-together-with-ai-center

[ref54] Vaishya R. Javaid M. Khan I. H. Haleem A. (2020). Artificial intelligence (AI) applications for COVID-19 pandemic. Diabetes Metab. Syndr. Clin. Res. Rev. 14, 337–339. doi: 10.1016/j.dsx.2020.04.012, PMID: 32305024 PMC7195043

[ref55] Voss C. A. (1985). The role of users in the development of applications software. J. Prod. Innov. Manag. 2, 113–121. doi: 10.1016/0737-6782(85)90007-4

[ref56] Xu D. Xu W. Kent M. Thomas L. Wang L. (2014). An automated test generation technique for software quality assurance. IEEE Trans. Reliab. 64, 247–268. doi: 10.1109/TR.2014.2354172

[ref57] Yatskiv S. Voytyuk I. Yatskiv N. Kushnir O. Trufanova Y. Panasyuk V. (2019). "Improved method of software automation testing based on the robotic process automation technology," in 2019 9th International Conference on Advanced Computer Information Technologies (ACIT). pp. 293–296.

